# Acute Subdural Haemorrhage as a Complication of Diagnostic Lumbar Puncture

**DOI:** 10.7759/cureus.7515

**Published:** 2020-04-02

**Authors:** Mai Abdullah, Ahmed Elkady, Areej Bushnag, Yehya Seddeq, Abdullah Alkutbi

**Affiliations:** 1 Neurology Department, International Medical Centre, Jeddah, SAU; 2 Neurology Department, Saudi German Hospital, Jeddah, SAU

**Keywords:** subdural haemorrhage, lumbar puncture, headache, cerebrospinal fluid, complication

## Abstract

Lumbar puncture (LP) is done daily for both spinal anesthesia and emergency cases for cerebrospinal fluid (CSF) analysis. Subdural hemorrhage (SDH) is a rare but severe complication following diagnostic LP, which could be potentially fatal, and early diagnosis may be life-saving. We present a 28-year-old female patient who presented to our emergency department with a headache, fever, and vomiting, with normal initial laboratory and imaging. Diagnostic lumbar puncture was done to exclude central nervous system infection. After CSF results and cultures were negative, and nasal influenza B detected, medication was started and she was discharged home. Three days post-procedure, she was complaining of a severe, persistent headache and a head computed tomography (CT) was done, which revealed acute SDH. Extensive workup searching for the cause of SDH was negative, and with a stationary course of SDH, she has discharged home again with a diagnosis of SDH post LP complication. In conclusion, a headache post LP is common and usually benign and self-limited. Severe persistent headache that is not positional and doesn't respond to analgesics with caffeine should be considered a red flag after LP and should be investigated carefully for other possibilities such as SDH.

## Introduction

Lumbar puncture (LP) is a frequent procedure in clinical practice. It is done daily either for the administration of spinal anesthesia or for obtaining cerebrospinal fluid (CSF) [1]. The common complications of LP are pain at the local site and post LP headache. Serious complications, such as subdural hemorrhage (SDH), brain stem herniation, and central nervous system (CNS) infection, could happen but, fortunately, it is infrequent [2]. The true incidence of SDH following LP is unknown because not all cases are reported and probably treated without investigation [3]. If any patient develops a severe headache after LP, which is not relieved in a supine posture with adequate hydration, SDH should be suspected, and the patient should be investigated and treated accordingly [4]. We report on a case of an uncommon SDH complication of an LP in a patient without any known risk factors for that.

## Case presentation

**Figure 1:**
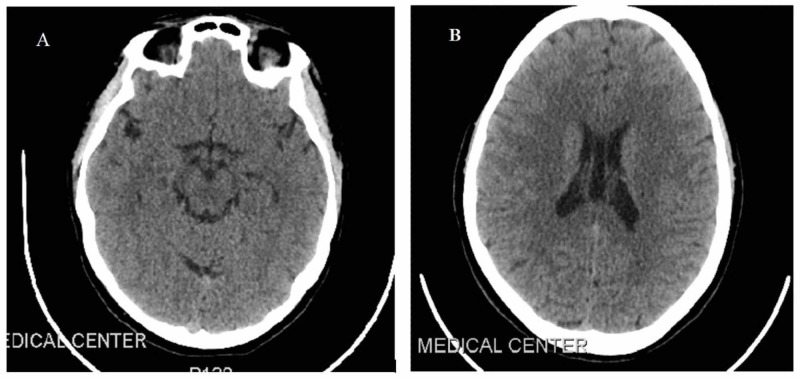
A, B: Non-contrast axial computed tomography brain on admission with no abnormalities

A 28-year-old female patient presented to the emergency department (ED) with a five-day history of high-grade fever, runny nose, severe persistent throbbing headache associated with nausea, vomiting (not projectile), and mild photophobia. Her vital signs showed 38.9 C temperature, 107/74 mm/Hg blood pressure, 115 beats/min pulse, soft, lax abdomen, and clear chest with equal air entry bilateral. On neurological examination, she was fully conscious, alert, oriented, with normal speech. Her neck was mildly stiff on flexion; however, Kerning and Brudzinski's signs were negative. There was tenderness of bilateral frontal sinus. The cranial nerves examination, including oculomotor nerves, visual acuity, and field, was normal. The fundus examination showed no papilledema. Other neurological examinations, including motor, sensory, and coordination, showed no abnormalities. A brain non-contrast computed tomography (CT) scan was done without any abnormalities detected (Figures 1A-1B). Initial serum laboratory investigations, including complete blood count, liver function test, renal function test, international normalized ratio (INR), and lactic acid, were normal, except that C-reactive protein (CRP) was 7.6 mg/L.

Moreover, rapid strep, dengue fever, and swine flu (H1N1) tests were negative, so a nasal swab and blood sample were sent for culture. A decision to perform LP to search for central nervous system (CNS) infection was made and written consent obtained from the patient. The procedure was done in the left lateral position by a senior anesthesiologist through dural puncture at the L3-4 vertebral level using a 22-gauge spinal needle. CSF opening pressure was 11 cmH2O. The fluid was clear, and three samples, each of 3 ml, were obtained and sent for the lab. CSF investigations were within normal ranges, including protein 0.19 gm/l, glucose 4.2 mmol/l, and white blood cell (WBC) count was 1/mm^3^. Moreover, gram staining showed no organism and culture pending. Over the 24 hours following the procedure, she was complaining of pain in the procedure site and post-LP positional headache, which responded to analgesics, caffeine, and proper hydration. On hospital day two, the nasal swab was positive for influenza B, so Tamiflu was started with an improvement of general condition. On day three, blood and CSF cultures were negative; the patient was afebrile and her headache improved, so she was discharged home with adequate pain killers and Tamiflu.

**Figure 2:**
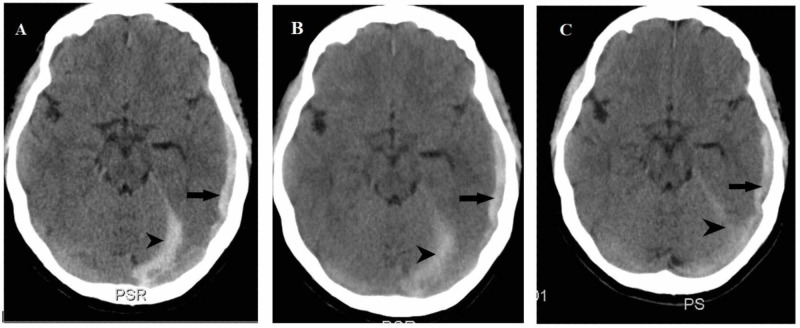
Non-contrast axial computed tomography brain (A) on readmission showing left temporal (black arrow) and left tentorium-cerebelli (arrowhead) subdural hemorrhage with stable course on day two (B) and decreased hemorrhage size of tentorium-cerebelli on day five (C)

Two days later, the patient came back to the emergency department (ED) complaining of drowsiness, a worse bilateral front-temporal headache not responding to analgesics and exacerbated by lying down and movement; moreover, it was associated with blurred vision, nausea, and vomiting. A new CT brain was done that revealed an acute left temporal SDH, extending within the tentorium-cerebelli with little mass effect (Figure 2A).

**Figure 3:**
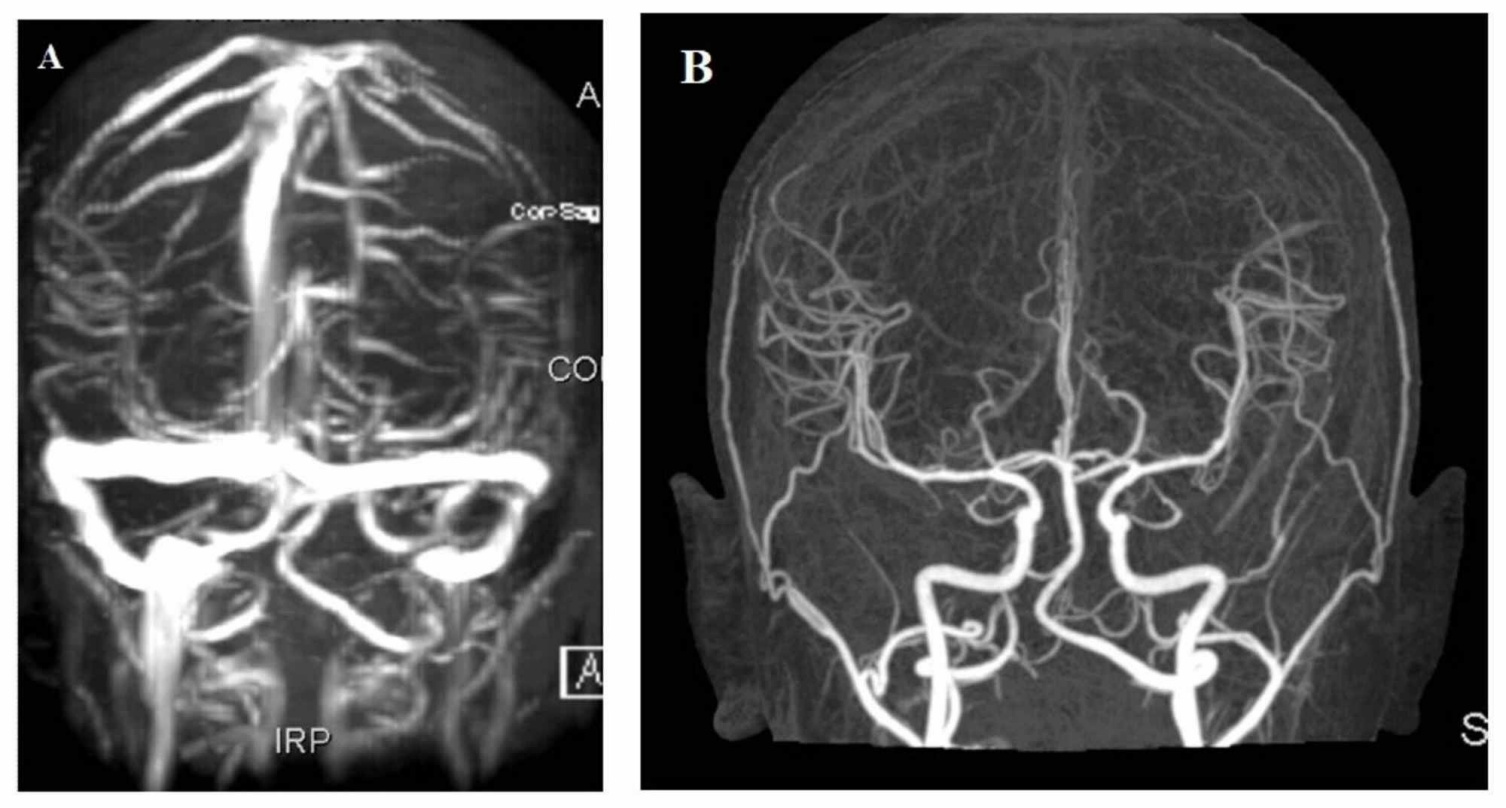
(A) Magnetic resonance venography with gadolinium contrast showed no evidence for dural venous sinus thrombosis, (B) CT cerebral angiography showed arteries are patent with no evidence for any aneurysms or arteriovenous malformation

The patient was admitted for close monitoring and symptomatic treatment. She denied any head trauma after discharge; extensive hematological workup for any bleeding tendency was negative. Magnetic resonance imaging (MRI) brain and magnetic resonance venography (MRV) with gadolinium (Figure 3A) was done and revealed the same hematoma and patent venous system without any signs of sinus thrombosis. Cerebral CT angiography (CTA) with contrast was done and didn't show any vascular malformation or aneurysmal dilatation (Figure 3B).

Neurosurgical consultation recommended conservative medical treatment and observation. She remained neurologically stable and her headache and general condition mildly improved. Repeat CT brain (Figure 2B) showed no change in SDH size and decreased hemorrhage size of tentorium-cerebelli on day five (Figure 2C). The clinical course was good, and the patient's headache gradually improved during the hospital course. She was discharged home on hospital day eight with analgesics and antiemetics with a diagnosis of post-LP SDH complication. She asked to follow up at the outpatient clinic and repeated CT brain after three weeks revealed decreased SDH size and entirely resolved SDH after two months.

## Discussion

SDH occurring after LP is extremely rare. Only sporadic case reports and a few small case series have described this condition [5-6]. We report on a case that experienced SDH post-diagnostic LP. The case readmitted with a headache, which is very common post-LP, however, post-lumbar puncture headache (PLPH) has a more benign, regressive course and responds well to analgesic and caffeine. The mechanism for PLPH is not fully understood, but continued CSF leakage, leading to a reduction in CSF pressure, is often blamed [7-8]. The international headache society (IHS) has developed a list of criteria to help differentiate PDPH from other, more serious, complications of dural puncture like SDH (Table 1) [9].

**Table 1:** International headache society criteria to diagnose a post-lumbar puncture headache CSF: cerebrospinal fluid, ICHD: international classification of headache disorders

Criteria of post-lumbar puncture headache according to ICHD-3
A. Headache has developed in temporal relation to the low CSF pressure or CSF leakage or led to its discovery.
B. Either or both of the following: 1. low CSF pressure (<60 mm CSF) 2. Evidence of CSF leakage on imaging
C. Dural puncture has been performed
D. Headache has developed within five days of the dural puncture
E. Not better accounted for by another ICHD-3 diagnosis

Although our patient headache met PLPH criteria and independent risk factors like female gender and middle age, there were red flags, for example, it was exacerbated by lying down, drowsiness, and non-responsiveness to fluids and medications. There have been other symptoms and signs considered red flags reported to be associated with SDH after LP at the time of diagnosis (Table 2) [10].

**Table 2:** Reported symptoms and signs with intracranial subdural hematoma caused by lumbar puncture.

Symptom/sign	Occurrence rate
Headache	74–91%
Nausea/vomiting	31–41%
Altered mental status	31–40%
Focal motor deficit	23–28%
Diplopia/visual changes	14–20%
Aphasia/dysarthria	11–13%

The development of SDH following LP is a rare but severe complication that can be fatal if left untreated (6). The true incidence of SDH following spinal anesthesia is not clear and mostly under-reported due to misdiagnosis as PLPH. However, one study estimated its occurrence as 1:220,000 [11]. Risk factors for the development of SDH after LP include large spinal needle size, multiple LPs, and large dural hole. Other risk factors in special populations are pregnancy, dehydration, use of anticoagulants, cerebral vascular abnormalities, and brain atrophy [4]. The primary pathophysiology of this complication is unknown; however, there are some data that it is the same as PLPH, with some minor differences. Intracranial hypotension allows a caudal shift of the brain, with traction on the arachnoid mater and dural veins; this leads to lesions of the blood vessels and could result in blood extravasations and formation of SDH [3,12].

## Conclusions

We report on a case that developed cranial SDH following diagnostic LP, which is a rare, yet serious, complication. Patients developing PDPH that is unrelieved by conservative measures, as well as a change of PDPH from postural to non-postural or an increase with lying down, require careful follow-up for the early diagnosis and management of possible SDH.
